# Association between dietary Intake, eating behavior, and childhood obesity among children and adolescents in Ethiopia

**DOI:** 10.1136/bmjnph-2021-000415

**Published:** 2023-11-15

**Authors:** Sibhatu Biadgilign, Tennyson Mgutshini, Bereket Gebremichael, Lioul Berhanu, Courtney Cook, Amare Deribew, Betemariam Gebre, Peter Memiah

**Affiliations:** 1 Department of Health Studies, College of Human Sciences, University of South Africa, Addis Ababa, Ethiopia; 2 Department of Health Studies, UNISA, Pretoria, South Africa; 3 Pediatrics and Child Health, Addis Ababa University College of Health Sciences, Addis Ababa, Ethiopia; 4 Nutrition Unit, Save the Children International, Addis Ababa, Ethiopia; 5 Department of Nursing, Fortis Institute - Pensacola, Pensacola, Florida, USA; 6 School of Public Health, Saint Paul’s Hospital Millennium Medical College, Addis Ababa, Ethiopia; 7 Nutrition International, Addis Ababa, Ethiopia; 8 Public Health Unit, International Medical Corps, Khartoum, Sudan; 9 Graduate School, University of Maryland, Baltimore, Maryland, USA

**Keywords:** Weight management, Dietary patterns, Nutrition assessment, Metabolic syndrome

## Abstract

**Introduction:**

The upsurge of overweight/obesity (OW/OB) among children and adolescents is as a result of complex interactions between lifestyle behaviours and socioeconomic factors. The objective of this study was to determine socioeconomic and sociodemographic factors, dietary intake and eating behaviours of children and adolescents in Ethiopia and their association with OW/OB.

**Methods:**

A cross-sectional study was conducted among 632 children and adolescents-parent dyads. To identify overweight/obese among children and adolescents, body mass index-for-age Z-scores by sex and age relative to WHO 2007 reference was calculated using WHO AnthroPlus software. A multivariable logistic regression model fitted to determine the adjusted associations between the outcome and the predictors selected from the bivariate analyses. Data analysis was carried out using STATA V.15.0.

**Results:**

The proportion of participants with low, medium and high dietary diversity scores was 7.28%, 22.5%, and 70.2%, respectively. Participants aged 13–18 years were less likely to be overweight or obese [adjusted OR (aOR) = 0.40; 95%CI: 0.26, 0.64] to those aged 5–12 years. Children in a family with the richest or highest socioeconomic status (SES) were more likely to be overweight or obese than those in families with the poorest or lowest status. Children and adolescents who consumed soft drinks (sugar-sweetened beverages) four or more times per week [aOR = 3.24; 95%CI: 1.13, 7.95] were more likely to be overweight or obese to those who did not consume soft drinks.

**Conclusions:**

The study identified factors such as younger age (<12 years), high SES and consumption of soft drinks as key contributors to overweight and obesity among children and adolescents. Therefore, interventions targeting behavioural prevention and reduction of overweight and obesity among children and adolescents should be cognizant of the above factors during implementation in order to achieve desired outcomes, further guided by exploratory qualitative studies to identify public perceptions and attitudes affecting dietary practices.

**WHAT IS ALREADY KNOWN ON THIS TOPIC**

- Most of the lifestyle factors like eating behaviours are modifiable and are associated with overweight and obesity in adolescents.
- The dietary intake of children leads to a higher intake of refined grains, animal-source foods, bulk intake of sugar and fats leading to a higher rate of obesity.
- Behaviours in school-aged children showed that the food habits of adolescents are characterised by high consumption of sweets and soft drinks and low consumption of fruits and vegetables.

**WHAT THIS STUDY ADDS**

- This observational cross-sectional study found that a large segment of overweight/obesity (OW/OB) (89.0%) children and adolescents consumed sugary snacks, about 68.1% of obese and 59.6% of non-obese children ate foodstuff while watching television/movies and 30.2% of obese and 22.4% non-obese children consumed soft drinks like Coca, Pepsi two times per week.
- The study also revealed that children having a family with high socioeconomic status, lower age group of the children and who drank soft drinks like Coca and Pepsi were more likely to be overweight/obese than their counterparts.

**HOW THIS STUDY MIGHT AFFECT RESEARCH, PRACTICE OR POLICY**

- This study highlights the ongoing need to focus on reducing the intake of sugar-sweetened beverages in order to reduce the prevalence of OW/OB.
- Policymakers should encourage and promote citizens to have healthy diets by increasing access to and provision of healthy and affordable foods and exploring other policy developments.

## Introduction

Globally, evidence demonstrated that morbidity and mortality related to overweight and obesity have doubled in the last two decades.^1^ Epidemiological studies have identified high body mass index (BMI) as a risk factor for chronic diseases, including cardiovascular disease, diabetes mellitus and chronic kidney disease.^2^ Available data indicates that there is a high burden of obesity observed among young adults in low/middle-income countries (LMICs). Early onset of obesity leads to a high cumulative incidence of type 2 diabetes and other cardiovascular diseases later in life.^1^

Childhood is a transitional period where eating behaviours and dietary patterns are established. Adolescence is also a critical period for the development of obesity and the foundation of chronic diseases and other health outcomes.^3^ Recent studies in 35 countries and regions in the WHO European Region and North America on health behaviours among school-aged children showed that the food habits of adolescents are characterised by high consumption of sweets and soft drinks, skipping breakfast and low consumption of fruits and vegetables.^4^ Globalisation, urbanisation, improved economic conditions and changing dietary intake in developing countries are risk factors contributing to rapid increase in obesity and other risk factors for non-communicable diseases (NCDs).^5 6^

The rise of overweight and obesity among children and adolescents is likely to be because of complex interactions among lifestyle behaviours, genetics; environmental characteristics surround the individual, dietary intake and socioeconomic factors.^7^ Lifestyle and behavioural factors such as eating behaviours, which are modifiable, are associated with overweight and obesity in adolescents.^8^ A systematic review of 15 studies conducted in Asian developing countries reported inconsistent statistical associations between different dietary behaviours and overweight/obesity (OW/OB) in children and adolescents.^9^

In most LMICs, there is a prominent shift of dietary intake characterised by higher intake of refined grains, animal-source foods, bulk intake of sugar and fats with minimal intake of fruits and vegetables.^10 11^ This has contributed to a higher rate of obesity and nutrition-related NCDs.^12^ Currently, there is limited evidence on the association of dietary intake and eating behaviour with childhood obesity in LMICs. This study therefore sought to assess the socioeconomic and sociodemographic factors, dietary intake and eating behaviours of children and adolescents in Ethiopia and their association with OW/OB.

## Methods

### Study design, population and setting

A cross-sectional study was conducted in the urban area of the municipality of Addis Ababa, Ethiopia. Addis Ababa is the capital city of Ethiopia and is sub-divided into 10 sub-city administrations. The sub-cities are further divided into several small administrative units called *woreda* (district). The details of the methods including sample size, sampling methods, inclusion and exclusion criteria are described elsewhere.^13^ The study population was paired sampled school-aged children with their mothers in the selected sub-cities during the study period. A total of 632 children and adolescents (99.6% response rate) who were living with their mothers in each of the sub-cities for at least 5 years, school going, aged 5–18 years old and those whose mothers gave consent were included. The study excluded children who were permanently ill whose caregivers/mothers were in a morbid state, severely ill (ie, not able to provide the necessary information) and in a state that was challenging to conduct or take any physical measurement (ie, scoliosis and kyphotic deformities).

As this paper is part of a larger study on obesity and food security, the study sample size was designed to estimate the point prevalence of obesity, and that no power calculations to detect differences in proportions among associated factors. The sample size was thus calculated using single proportions sample size formula generated using Epi Info statistical package (Centers for Disease Control and Prevention, Atlanta, USA, 2010). The following parameters were used to calculate the sample size: proportion of children who were overweight in the population (P) is 9.5 %,^14^ 95% CI [Z-The standard normal value at (100%−α) confidence level], d-3% of margin of error for sampling and 80% power. This gave a sample size of 367. A 15% non-response rate and design effect of 1.5 were included and this generated a total representative sample size of 634. Multi-stage sampling technique was carried out to identify the study unit from selected sub-cites. Proportion to population sampling was applied to obtain the sample size in each sub-city. Thereafter, simple random sampling method was used to select districts and Kebeles (the smallest administrative unit in the government structure) in each sub-city. The study was conducted in five sub-cities in Addis Ababa namely, Bole, Gulele, Kolfe Keranio, Nifasilk Lafto and Yeka. All Ethiopian population groups are represented within Addis Ababa due to its position as the capital of the country. Data collection period was from May to July 2017.

### Data collection instruments and procedures

The study used structured and pretested questionnaires that were administered to mothers in their households (see online supplemental file 1). The questionnaire was adopted from a validated instrument developed in English and then translated into Amharic. The tool was then retranslated back to English by another person to verify its consistency and flow. The content of the questionnaire included: sociodemographic characteristics (child age and sex; age, educational status, and occupational status of the mother); socioeconomic status (SES)/wealth index; dietary intake; and child nutritional status indicators based on anthropometric measurements. Pretesting of the questionnaire was carried out on 10% of the final sample size in a similar area before actual data collection took place. For the dietary intake assessment, dietary intake was collected via a single 24-hour dietary recall (24HR). A 24HR is a structured interview intended to capture detailed information about all foods and beverages consumed by the participants in the past 24 hours, most commonly from midnight to midnight the previous day. The dietary intake data collected through the 24-hour recalls was combined into nine food groups ((1) basic staples, (2) vitamin A-rich fruits and vegetables, (3) other fruits, (4) other vegetables, (5) legumes and pulses, (6) meat or fish; (7) oil; (8) dairy; and (9) eggs) based on the Food and Agriculture Organization/Food^15^ and Nutritional Technical Assistance Household Dietary Diversity Questionnaire and Guidelines. The data collection team consisted of one supervisor, four females, and two male interviewers. Similarly, data collectors were well-qualified health professionals who practice in the health facilities in Addis Ababa and some of them attended postgraduate programs at Addis Ababa University at the levels of Master and PhD. At analysis, participants were divided into three groups, including low, medium, and high dietary diversity, based on the dietary diversity scores (DDS). The DDS were calculated based on food groups consumed and grouped into low if the DDS group was ≤3, medium if DDS was 4 and 5, and high if DDS group participants have DDS ≥6.^16^

10.1136/bmjnph-2021-000415.supp1 Supplementary data

### Anthropometric data

Height and weight measurements were assessed among children and adolescent in all selected households. Weight measurement was taken using lightweight, SECA mother-infant scales with a digital screen designed and manufactured under the guidance of the UNICEF. Height measurement was assessed using a standing measuring board. Weight and height of each child were recorded to the nearest 0.1 kg and 0.1 cm, respectively. Height and weight measurements of children were converted into Z-scores based on the WHO reference population considering their age and sex. The BMI-for-age Z-scores by sex and age among children and adolescents relative to WHO 2007 reference was calculated using WHO AnthroPlus software to identify for overweight/obese.^17^ The outcome variable used for analysis in this study was childhood OW/OB as a binary variable and defined as more than 1 SD above the median based on WHO growth reference.^18^ The research team secured informed consent from responding mothers and children especially considering sensitive nature anthropometric data.

### Statistical analysis

Descriptive statistics were used to characterise the study population and other several variables in terms of frequency/percentages for categorical variables and means and SD for continuous variables. χ^2^ test was applied to identify relationships between overweight/obese children and adolescents and several of the background, household, child and maternal characteristic. Explanatory variables at the bivariate analysis, which showed association with the outcome at p<0.2, were included in the final logistic regression models.^19 20^ A multivariable logistic regression model with robust estimations of SEs was fitted to determine the adjusted associations between the outcome and the predictors selected from the bivariate analyses (socioeconomic and sociodemographic factors, dietary intake and eating behaviour). In the final model, associations with a p<0.05 were considered statistically significant. Data analysis was carried out using STATA V.15.0 (Stata Corporation, College Station, TX) and WHO AnthroPlus software V.1.02 (WHO, Geneva, Switzerland).

### Patient and public involvement

There was no direct patient or public involvement in the setting of the research questions or outcome measure, design, recruitment and conduct, reporting and dissemination plans of this study.

## Results

### Characteristics of the study subjects

A total of 632 children participated in the study, with 48% being male. Half, or 322 (51%) of the children were within the age group of 13–18 years. The mean age was 12.5 (±2.96) years. Regarding marital status, 84.2% of participants’ mothers were married (table 1).

**Table 1: Participants':** sociodemographic characteristics among children and adolescents according to overweightorobesity status

Characteristics/variables	Categories	Childhood overweight/obesity		P value
		Yes, n (%)	No. 1 (%)	
Age group of the children and adolescents	5–12 years	108 (59.3)	202 (44.9)	0.001
	13–18 years	74 (40.7)	248 (55.1)	
Sex of children and adolescents	Male	85 (46.7)	221 (49.1)	0.583
	Female	97 (53.3)	229 (50.9)	
Sex of household head	Male	140 (76.9)	341 (75.8)	0.760
	Female	42 (23.1)	109 (24.2)	
Age group of household head	<40 years	22 (30.2)	176 (39.1)	0.036
	≥40 years	127 (69.8)	274 (60.9)	
Maternal education	Illiterate	63 (34.6)	180 (40)	0.208
	literate	119 (65.4)	270 (60)	
Maternal occupation	Unemployed	50 (27.5)	161 (35.8)	0.011
	Private business	30 (16.5)	96 (21.3)	
	Employed	102 (56.0)	193 (42.9)	
The marital status of the respondents	Married	153 (84.1)	379 (84.2)	0.747
	Divorced	17 (9.34)	33 (7.3)	
	Widowed	10 (5.49)	32 (7.1)	
	Separated	2 (1.10)	6 (1.3)	
Household wealth index	Poorest	28 (15.6)	98 (22.1)	<0.001
	Poorer	32 (17.8)	92 (20.7)	
	Middle	35 (19.4)	117 (26.3)	
	Richer	31 (17.2)	67 (15.1)	
	Richest	54 (30.0)	70 (15.8)	

### Household dietary diversity

The mean (SD) DDS of the household was 6.63 (±2.00), with 7.8% low, 22.2% medium and 70.0% high DDSs. About 98% of the participants consumed starchy staple foods, including a combination of cereals and white roots and tubers, in 24 hours. More than 80% of participants ate dark green leafy vegetables, vitamin A-rich fruits and vegetables and other fruits and vegetables. The consumption of organ meat was lower than the rest of the food groups (table 2). This study showed that 57.7% and 53.9% of overweight or obese participants at the household and individual level mentioned consuming meals or snacks outside the home on the day before the interview (see Table 3).

**Table 2: Food:** groups consumed for children and adolescents in the last 24 hours preceding the study

Food groups	Frequency	Per cent
Starchy staples*	622	98.4
Dark green leafy vegetables	517	81.8
Other vitamin-rich fruits and vegetables	527	83.4
Other fruits and	549	86.9
Organ meat	210	33.2
Meat and fish§	401	63.4
Eggs	396	62.7
Legumes, nuts and seeds	479	75.8
Milk and milk products	491	77.7

*The starchy staples food group is a combination of cereals, white roots and tubers.

†The other vitamin A-rich fruit and vegetable group is a combination of vitamin A-rich vegetables and tubers and vitamin A-rich fruits.

The other fruit and vegetable group is a combination of other fruits and other vegetables.

The meat group is a combination of meat and fish.

**Table 3: Dietary:** habits and eating behaviours-related factors associated with overweightorobesity among children and adolescents

Variables	Categories	Childhood overweight/obesity		P value
		Yes, n (%)	No. 1 (%)	
Dietary diversity score (DDS) category	Low (DDS ≤3)	35 (7.8)	11 (6.0)	0.744
	Medium (DDS 4 and 5)	100 (22.2)	42 (23.1)	
	High (DDS ≥6)	315 (70.0)	129 (70.9)	
Did you or anyone in your household eat anything (a meal or snack) outside the home yesterday?	Yes	105 (57.7)	233 (51.8)	0.177
	No	77 (42.3)	217 (48.2)	
Did you eat anything (a meal or snack) outside the home yesterday?	Yes	98 (53.9)	214 (47.6)	0.152
	No	84 (46.1)	236 (52.4)	
How many days per week do you usually eat fruits such as oranges and bananas?	No intake	89 (19.8)	28 (15.4)	0.102
	One day	184 (40.9)	66 (36.3)	
	Two days and above	177 (39.3)	88 (48.3)	
How many days per week do you usually eat vegetables?	No intake	26 (14.3)	72 (16.0)	0.749
	One day	60 (33.0)	133 (29.6)	
	Two days	62 (34.1)	149 (33.1)	
	Three days and more	34 (18.7)	96 (21.3)	
Do you have a snack today?	Yes	162 (89.0)	406 (90.2)	0.648
	No	20 (11.0)	44 (9.8)	
How many times a day do you have a snack?	One time	119 (73.5)	322 (79.3)	0.150
	Two times	28 (17.3)	63 (15.5)	
	Three times and more	15 (9.2)	21 (5.2)	
How often do you serve meals per day other than snacks?	One time	3 (1.8)	23 (5.7)	0.210
	Two times	4 (2.5)	15 (3.7)	
	Three times	148 (91.4)	352 (86.7)	
	Four times and more	7 (4.3)	16 (3.9)	
How do you get your lunch?	Bring from home	154 (84.6)	397 (88.4)	0.607
	Buy from school cafeteria	23 (12.6)	41 (9.1)	
	Buy from nearby food service establishment	1 (0.6)	2 (0.5)	
	I did not use lunch	4 (2.2)	9 (2.0)	
Types of foods that you usually bring to school in addition to the regular meal	Cake	37 (20.3)	111 (24.7)	0.117
	Biscuit	90 (49.5)	228 (50.8)	
	Ice cream	15 (8.2)	22 (4.9)	
	Chocolate	26 (14.3)	42 (9.4)	
	Others	14 (7.7)	46 (10.2)	
Do you eat when you watch television or movies?	Yes	124 (68.1)	267 (59.6)	0.135
	No	54 (29.7)	168 (37.5)	
	I did not watch television or movies	4 (2.2)	13 (2.9)	
Do you eat food when you study?	Yes	66 (36.3)	146 (32.6)	0.376
	No	116 (63.7)	302 (67.4)	
How often do you drink soft drinks per week?	I do not drink these	36 (19.8)	127 (28.2)	<0.001
	One time	53 (29.1)	151 (33.6)	
	Two times	55 (30.2)	101 (22.4)	
	Three times	12 (6.6)	50 (11.1)	
	Four times and more	26 (14.3)	21 (4.7)	
A child eats in school	Yes	146 (80.2)	346 (76.9)	0.361
	No	36 (19.8)	104 (23.1)	
A child carries packed lunch	Yes	163 (89.6)	398 (88.4)	0.687
	No	19 (10.4)	52 (11.6)	
A child buys snacks	Yes	93 (51.1)	186 (41.3)	0.025
	No	89 (48.9)	264 (58.7)	

### Children’s dietary habits and eating behaviours

A high proportion of OW/OB (89.0%) children and adolescents consumed snacks within 24 hours of data collection. Likewise, 17.3% and 9.2%; and 15.5% and 5.2% of OW/OB and non-OW/OB children and adolescents consumed snacks two times, three times and more, respectively. In addition, 68.1% and 59.6% of OW/OB and non-OW/OB children, respectively ate foodstuff while watching television/movies. Approximately about 30.2% of OW/OB and 22.4% non-OW/OB children consumed soft drinks two times per week. Approximately 39.3% of (OW/OB) children and adolescents ate fruits such as orange, banana more than 2 days and above per week. Similarly, only 18.7% of OW/OB children and adolescents ate vegetables more than 3 days and above per week (see table 3). The differences between the eating behaviours in OW/OB and non-OW/OB were not statistically significant based on the results of table 3.

### Factors associated with OW/OB among children and adolescents

At bivariate logistic regression models, the following variables were significantly associated with OW/OB (p<0.05): age group of children and adolescents, age group of household head, maternal education, maternal occupation and household wealth index, intake of soft drinks, days per week where you usually eat fruits, frequency of having a snack in a day, frequency of serving meals per day other than snack, type of foods that you usually bought to school in addition to the regular meal, eating while watching television/movies and child buying snacks. At multivariable logistic regression, participants aged 13–18 years were less likely to be overweight/obese (adjusted OR (aOR)=0.40; 95% CI 0.26, 0.64) compared with those aged 5–12 years.

Children in a family with richest/high SES (aOR=3.28; 95% CI 1.49, 7.20) were more likely to be overweight/obese than those in families of poorest/low SES. However, there was no clear evidence of a ‘gradient’ on the basis of the OR. Children and adolescent who drank soft drinks for four or more times per week (aOR=3.24; 95% CI 1.13, 7.95) were more likely to be overweight/obese compared with those who did not drink (see table 4).

**Table 4: Factors:** associated with overweightorobesity among children and adolescents

Characteristics/variables	Categories	Crude Ratio	Adjusted Ratio
Age group of the children and adolescents	5–12 years	Ref	Ref
	13–18 years	0.56 (0.39, 0.79)	0.40 (0.26, 0.64)
Age group of household head	<40 years	Ref	Ref
	≥40 years	1.48 (1.03, 2.14)	1.30 (0.81, 2.07)
Maternal education	Illiterate	Ref	Ref
	literate	1.26 (0.88, 1.80)	1.00 (0.62, 1.63)
Maternal occupation	Unemployed	Ref	Ref
	Private business	1.00 (0.60, 1.69)	0.80 (0.40, 1.47)
	Employed	1.70 (1.14, 2.53)	1.60 (0.96, 2.67)
Household wealth index	Poorest	Ref	Ref
	Poorer	1.47 (0.80, 2.69)	1.77 (0.87, 3.64)
	Middle	1.08 (0.58, 2.00)	1.28 (0.60, 2.73)
	Richer	1.62 (0.88, 2.97)	1.51 (0.67, 3.38)
	Richest	3.14 (1.75, 5.64)	3.28 (1.49, 7.20)
How many days per week do you usually eat fruits such as oranges and bananas?	No intake	Ref	Ref
	One day	1.14 (0.68, 1.89)	0.84 (0.37, 1.56)
	Two days and above	1.58 (0.96, 2.59)	1.03 (0.44, 2.24)
How many times a day do you have a snack?	One time	Ref	Ref
	Two times	1.20 (0.73, 1.97)	0.80 (0.41, 1.70)
	Three times and more	1.93 (0.96, 3.87)	1.03 (0.46, 2.31)
How often do you serve meals per day other than snacks?	One time	Ref	Ref
	Two times	2.04 (040, 10.4)	2.05 (0.34, 12.4)
	Three times	3.22 (0.95, 10.9)	3.24 (0.88, 11.9)
	Four times and more	3.35 (0.75, 14.9)	2.86 (0.57, 14.3)
List foods that you usually bring to school in addition to the regular meal	Cake	Ref	Ref
	Biscuit	1.18 (0.76, 1.85)	1.17 (0.67, 2.05)
	Ice cream	2.04 (0.96, 4.35)	1.49 (0.60, 3.73)
	Chocolate	1.86 (1.00, 3.43)	1.48 (0.71, 3.07)
	Others	0.91 (0.45, 1.85)	0.96 (0.38, 2.42)
Do you eat when you watch television or movies?	Yes	Ref	Ref
	No	0.69 (0.47, 1.00)	0.79 (0.48, 1.28)
	I did not watch television or movies	0.66 (0.21, 2.07)	1.24 (0.34, 4.62)
How often do you drink soft drinks per week?	I do not drink these	Ref	Ref
	One time	1.24 (0.76, 2.01)	1.58 (0.84, 2.98)
	Two times	1.92 (1.17, 3.15)	1.36 (0.69, 2.74)
	Three times	0.85 (0.41, 1.76)	0.56 (0.20, 1.35)
	Four times and more	4.37 (2.20, 8.65)	3.24 (1.13, 7.95)
A child buys snacks	Yes	Ref	Ref
	No	0.67 (0.48, 0.95)	0.96 (0.60, 1.52)

## Discussion

This study aimed to assess dietary intake and eating behaviours of children and adolescents in Ethiopia and their association with overweight and obesity. The study found that SES and intake of sugar-sweetened beverages were positively associated with OW/OB. Younger age was associated with a higher likelihood of being OW/OB. There was no association between DDS and the risk of being overweight/obese.

Children’s age was an important factor associated with OW/OB. In this study, respondents from 13 to 18 years were less likely to be overweight/obese as compared with 5–12 years. Similarly, data obtained from the National Health and Nutrition Examination Surveys in the USA demonstrated that obesity increased from 11.3% in 1988–1994 to 19.6% in 2007–2008 during the adolescent age among children aged 6–11 years.^21^

Another study in Egypt concluded that childhood BMI had a significant positive correlation with the age of the child among Egyptian children.^22^ A study using data from a population-based cohort in Norway showed that prevalence of obesity increased in children with age.^23^ Similar findings were reported from school children in Central Ethiopia where older children were about 6.7 times more likely to be overweight and/or obese compared with younger ones.^24^ Another study from the USA documented a significant increase in obesity and severe obesity with age, with a sharp increase among preschool-aged children (children aged 2–5 years).^25^

In contrast to findings of these studies, the prevalence of OW/OB in our study was established to be lower in the adolescent age group compared with children. This difference could be due to the cultural influences. For example, commonly observed culture or practice of buying sweet beverages, fast foods and highly processed energy dense foods for young children in most particularly better-off households or high SES, which may be a modifiable and contextual factor that merits further qualitative assessment.

Researches in LMIC showed increased availability of energy-dense, nutrient-poor, ultra-processed foods is potentially detrimental to adolescent diets and nutritional status.^26 27^ In addition to this, there are significant biological changes in children and adolescents between the age groups of 5–12 years and 13–18 years. This is predominantly due to the preparation for adolescent growth spurt and the growth spurt itself that are likely to influence OW/OB classification.^28^ However, we recommend that researchers consider longitudinal studies to observe the effects of children’s age and OW/OB in specific populations group.

The findings of this study revealed that children consuming soda frequently were almost four times more likely to be overweight/obese compared with those who did not consume soft drinks. In line with these findings, a systematic review conducted in Asian countries demonstrated that OW/OB in children and adolescents was connected with high intake of fast food, presence of snacking and drinking sugar-sweetened beverages.^9^ Similar findings were also reported in other studies done in different countries.^29–31^ This can be explained by the fact that greater number of sugary beverages a person has each day, the more calories he or she takes which leads to positive energy balance resulting in obesity. However, it is difficult to make direct comparisons between studies because sugar content in sweetened beverage consumptions varies from country to country.

Again, those children from higher socioeconomic families (wealth index) have an increased tendency to have children and adolescents who are overweight or obese. The justification is that higher classes in developing countries would probably have access to plenty of high-calorie foods. This is similar to families with lower status (SES) in developed countries. This may drive weight gain, which is the core element for the high prevalence and development of overweight and obesity in higher wealth index families in developing countries.^32^ Similar to our finding, studies showed high wealth category is linked to higher prevalence of overweight/obesity (OW/OB).^33–37^ This implies that there is evidence of LMICs undergoing economic, epidemiological, and nutritional transitions.^38^ However, there is a shift from strata of low-income status (SES) to strata of middle-income status (SES) in the majority of LMICs. Overweight and obesity among children were more prevalent in the highest socioeconomic groups.^39^ In addition to the above, mostly children and adolescents from higher socioeconomic groups are characterized by consumption of high-fat and high-calorie diets accompanied by a more sedentary lifestyle. Similar findings in Ethiopia and other developing countries might be due to the perception of childhood and adolescent overweight and obesity as signs of being healthy, although such assertions require further exploration.

The strengths of this study include the high participation rate and the inclusion of several factors related to dietary intake and eating behavior. The weaknesses include the possibility of recall and social desirability bias due to the use of self-reports and retrospective dietary recall. Every possible effort was made during the interview to minimize recall bias in reporting diverse food groups consumed over the previous day. This was done by limiting the recall period to no more than a month for food and dietary questions and by informing the participants of the study. The other limitation is the use of a single 24-hour recall dietary assessment. A single 24-hour recall is not enough to describe an individual’s usual intake of food and nutrients and their true dietary pattern in one measurement. The study also did not assess the daily caloric intake of the study participants, which could have an effect on childhood obesity. The cross-sectional nature of the study does not allow for any inference of causality to be made. Similarly, the cultural and social dynamics in terms of the sedentary lives of people brought by ‘modern’ habits like watching TV influence the dietary intake (sodas and sweetened beverages) and would be more important to be seen from a qualitative study perspectiv. Additional sources of bias might occur in the selection of participants (inclusion/exclusion criteria), for example, limiting participation to those residents for >5 years.

In conclusion, the study indicates that SES and intake of sugar-sweetened beverages were positively associated with OW/OB. Lower age was associated with a higher likelihood of being OW/OB. There was no association between DDS and the risk of OW/OB. The study has identified possible factors to consider when planning for obesity prevention interventions among children and adolescents in the study settings. This could include the reduction of the consumption of sugar-sweetened beverages. It will be more relevant to undertake a qualitative study to understand the cultural and social dynamics related to the fact to have OW/OB as well as further guided by exploratory qualitative studies to identify public perceptions and attitudes affecting dietary practices. Assessing parental and students’ awareness on the issue, availability/affordability of healthy diet, the school environment and intervene accordingly is recommended.

## Data Availability

Data are available upon reasonable request.
