# Supplementary material for: Association between dietary Intake, eating behavior, and childhood obesity among children and adolescents in Ethiopia
Source: BMJ Nutr Prev Health. 2023 Nov 15;6(2):203–11. doi: 10.1136/bmjnph-2021-000415 (PMC11009522; doi:10.1136/bmjnph-2021-000415)
Supplement: Supplementary data [file bmjnph-2021-000415supp001.pdf]

Annexure: Structured Questionnaire English Version Used for Data Collection

SECTION I- SOCIO-DEMIOGRAPHIC CAHACTERSTICES

Sr.N	Questions and filters	Coding categories	Skip
101	Age of child	__________	
102	Child's sex	1. Boy<br>2. Girl	
103	Sex of household head	1. Male<br>2.Female	
104	Age of household head	________________	
105	Educational status of the mother	1. Unable to read and write<br>2. Read/write but no formal education.<br>3.If attended formal education; highest grade completed	
106	Occupational status of the mother	1. Farmer<br>2. Merchant<br>3. Governmental employee<br>4. Non-governmental employee<br>5. Daily laborer<br>6. Jobless<br>99. Others (specify)______	
107	Marital status of the mother	1. Married<br>2. Divorced<br>3. Widowed<br>4. Separated	
108	Does your household have:<br>Electricity?<br>Radio?<br>Television?<br>Telephone?<br>Personal computer?<br>Internet?<br>Refrigerator?<br>A table?<br>A chair?	Yes No<br>1. Electricity? 1 2<br>2. Radio? 1 2<br>3. Television? 1 2<br>4. Telephone? 1 2<br>5. Personal computer? 1 2<br>6. Internet? 1 2<br>7. Refrigerator? 1 2<br>8. A table? 1 2<br>9. A chair? 1 2	

109	Does any one of your household member own:	Yes No<br>1. A bajaji/motorcycle? 1 2<br>2. An animal-drawn cart? 1 2<br>3. A car/truck? 1 2<br>4. A bicycle? 1 2	
110	How many rooms in this household are used for sleeping?	ROOMS__________________	
111	Main material of the floor(observe)<br>[Finished-polished wood, asphalt, ceramic, cement & carpet]	1. Natural (earth/sand)<br>2. Rudimentary (wood/bamboo)<br>3. Finished floor<br>99. Other (specify)	
112	Main material of the roof (observe)<br>[Finished roofing-corrugated iron/metal, wood, cement, concrete]	1. Natural roofing (no roof/leaf)<br>2. Rudimentary (plastic/bamboo/wood)<br>3. Finished roofing<br>99. Other (specify)____________	
113	Main material of the exterior wall (observe)<br>[Natural wall- if no wall, orcane/trunks/bamboo/reed or dirt]	1. Natural wall (no wall/reeds/dirt)<br>2. Rudimentary wall<br>3. Finished wall<br>99.Other (specify) ___________	
114	What is the main source of drinking water for your household?	1. Tap water (piped into house or yard)<br>2. Protected dug well/spring/rain water<br>3. Public tap<br>99.Other (specify) ____________	
115	What kinds of toilet facility do members of your household use?	1. Pour flush latrine (private or shared)<br>2. Pit latrine (private or shared)<br>3. Public toilet<br>99. Other (specify)____________	
116	Does any member of this household have a bank or microfinance saving account?	1. Yes<br>2. No	

SECTION II- dietary diversity

Now I would like to ask you about the types of foods that you ate yesterday during the day and at night/ During the privies 24-hour period (The time from this time yesterday until know) did you consumed any of the following food types:

Interviewer: Mark ''Yes'' if the respondent eats even one of the listed food examples.

Sr.N	Food group	Examples	Response
201	Cereals	Bread, 'ambasha', rice noodles, biscuits, cookies, or any other foods made from millet, sorghum, maize, rice, wheat,	1. YES<br>2. NO
202	White tubers and roots	White potatoes, white yams, cassava, or foods made from roots.	1. YES<br>2. NO
203	Vitamin A rich vegetables and tubers	Pumpkin, carrot, squash, or sweet potato that are orange inside + other locally available vitamin A rich vegetables (e.g. red sweet pepper)	1. YES<br>2. NO
204	Dark green leafy vegetables	Any dark, green, leafy vegetables such as bean leaves, kale, spinach, Sweet pepper, pepper leaves, taro leaves, and amaranth leaves including wild ones.	1. YES<br>2. NO
205	Other vegetables	Any other vegetables (e.g. tomato, onion, eggplant) including wild vegetables + other locally available vegetables	1. YES<br>2. NO
206	Vitamin A rich fruits	Ripe, mangoes, orange, papayas apricot (fresh or dried), dried peach, and 100% fruit juice made from these + other locally available vitamin A rich fruits	1. YES<br>2. NO
207	Other fruits	Any other fruits, including wild fruits and 100% fruit juice made from these	1. YES<br>2. NO
208	Organ meat (iron-rich)	liver, kidney, heart or other organ meats or blood-based foods	1. YES<br>2. NO
209	Flesh meat products	Beef, pork, lamb, goat, rabbit, wild game, chicken, duck, or other birds, insects	1. YES<br>2. NO
210	Eggs	Any eggs? eggs from chicken, duck, guinea fowl or any other egg	1. YES<br>2. NO
211	Fish	Any fresh or dried fish or shellfish?	1. YES<br>2. NO
212	Legumes, nuts and seeds	Any beans, peas, lentils, nuts, seeds or foods made from these(eg. hummus, peanut butter)	1. YES<br>2. NO
213	Milk and milk products	Any cheese, yogurt, milk or other milk products?	1. YES<br>2. NO
214	Oils and fats	Any foods made with oil, fat, or butter added to food or used for cooking?	1. YES<br>2. NO
215	sweets	Any sugar, honey, sweetened soda or sweetened juice drinks, sugary foods such as chocolates, candies, cookies and cakes	1. YES<br>2. NO
216	spices, condiments, beverages	spices (black pepper, salt), condiments (soy sauce, hot sauce), coffee, tea, alcoholic	1. YES<br>2. NO

		beverages	
217	Household level only	Did you or anyone in your household eat anything (meal or snack) OUTSIDE the home yesterday?	1. YES<br>2. NO
218	Individual level	Did you eat anything (meal or snack) OUTSIDE the home yesterday?	1. YES<br>2. NO

SECTION III- Child Dietary Pattern and Habits

Sr.N	Questions and filters	Coding categories	Skip
301	List three foods that you usually eat	1………………………….<br>2……………………………..<br>3……………………………….	
302	List three foods that you do not usually eat	1………………………………….<br>2…………………………….<br>3………………………………..	
303	How many days per week did you usually eat fruit such as orange, banana	1.No intake<br>2.One day<br>3.Two days and above	
304	How many days per week did you usually eat vegetables?	1.No intake<br>2.One day<br>3.Two days<br>4.Three days and above	
305	Do you have a snack currently?	1.Yes<br>2.No, if no go to Q409	
306	If your answer is yes, what do you have for snack?	………………………..	
307	How many times a day do you have snack?	1.One times<br>2.Two times<br>3.Three times and above	
308	How often do you serve meals per day other than snack?	1.One times<br>2.Two times<br>3.Three times<br>4.Four and above	
309	How do you get your lunch?	1-Bring from home<br>2-Buy from school cafeteria<br>3-Buy from nearby food service establishment.<br>4-I did not use lunch	
310	List foods that you usually bought in addition to the regular meal	1-cake<br>2-biscuit<br>3-ice cream<br>4-chocolate<br>99-others (specify)……….	

311	Do you eat when you watch television/movies?	1-yes<br>2-no<br>3- I didn't not watch television/movies	
312	Do you eat food when you study?	1-Yes<br>2- No	
313	How often do you drink soft drinks like Coca, Pepsi per week?	1- I do not drink<br>2-One times<br>3-Two times<br>4-Three times<br>5- Four and above	
314	Child eats in school	1-Yes<br>2- No	
315	Child carries packed lunch	1-Yes<br>2- No	
316	Child buys snacks	1-Yes<br>2- No	

SECTION IV- Physical Measurements

**Anthropometric measurements of the child**			
**Sr.N**	**Questions and filters**	**Coding categories**	**skip**
401	Height of the child	_______________cm	
402	Weight of the child	_______________kg	
